# C-Reactive Protein Level at Time of Discharge Is Not Predictive of Risk of Reoperation or Readmission in Children With Septic Arthritis

**DOI:** 10.3389/fsurg.2019.00068

**Published:** 2019-12-03

**Authors:** Maryse Bouchard, Lara Shefelbine, Viviana Bompadre

**Affiliations:** ^1^Division of Orthopaedics, The Hospital for Sick Children, Toronto, ON, Canada; ^2^School of Medicine, University of Washington, Seattle, WA, United States; ^3^Division of Orthopaedics and Sports Medicine, Seattle Children's Hospital, Seattle, WA, United States

**Keywords:** reoperation, readmission, C-reactive protein, pediatric, septic arthritis (SA)

## Abstract

**Purpose:** C-reactive protein (CRP) level is used at our tertiary pediatric hospital in the diagnosis, management, and discharge evaluation of patients with septic arthritis. The purpose of this study was to evaluate the efficacy of a discharge criterion of CRP < 2.0 mg/dL for patients with septic arthritis in preventing reoperation and readmission. We also aimed to identify other risk factors of treatment failure.

**Methods:** Patients diagnosed with septic arthritis between January 1, 2007 and December 31, 2017 were identified with ICD 9/10 and related CPT codes. Systematic chart reviews were performed to obtain demographic data, infection characteristics, and treatment details. Bivariate tests of associations between potential risk factors and readmission and reoperation were performed. Quantitative variables were analyzed using Mann-Whitney tests and categorical variables were analyzed using Chi-square tests.

**Results:** One hundred and eighty-three children with septic arthritis were included in the study. Seven (3.8%) were readmitted after hospital discharge for further management, including six who required reoperation. Mean CRP at discharge for single-admission patients was 1.71 mg/dL (± 1.07) and 1.96 mg/dL (± 1.19) for the readmission group (*p* = 0.664). Forty-eight children (25.9%) had CRP values greater than the recommended 2.0 mg/dL at discharge. Only three of these patients (6.2%) were later readmitted. The only common variable of the readmitted children was an antibiotic-resistant or atypical causative bacteria.

**Conclusions:** CRP levels are useful in monitoring treatment efficacy of septic arthritis in children but are not reliable as a discharge criterion to prevent readmission or reoperation. We recommend determining discharge readiness on the basis of clinical improvement and down-trending CRP values. There was a higher risk of readmission in children with an antibiotic-resistant or atypical causative bacteria. Close monitoring of these patients and those with negative cultures at time of discharge is suggested to identify signs of persistent infection.

**Level of evidence:** III, retrospective cohort study.

## Introduction

Septic arthritis is a serious condition that accounts for 21% of acute pediatric musculoskeletal infections (1). Sequela range from growth disturbance, arthritic changes, avascular necrosis, and pathologic fracture to potentially life-threatening conditions such as septic thrombophlebitis, sepsis, and death (1–9). Diagnosis of septic arthritis is suspected based on clinical history, physical exam, laboratory results (CBC with differential, C-reactive protein (CRP), and erythrocyte sedimentation rate (ESR) and imaging (ultrasound or MRI), with confirmation by aspiration or tissue culture from the affected joint (10).

The recommended treatment protocol for septic arthritis at our tertiary pediatric hospital is surgical irrigation and debridement, post-operative intravenous (IV) antibiotics until CRP is < 2.0 mg/dL followed by oral antibiotics for a total of 4 weeks. CRP is a rapidly responsive and sensitive marker of inflammation. Kallio et al. (11) noted that if CRP is decreasing, inflammation is likely to be subsiding even if the clinical symptoms and signs of the patient have not yet started to diminish. Other studies have demonstrated the usefulness of trending CRP when assessing resolution of infection in septic arthritis (12, 13). Negative predictive value of CRP ranges from 78 to 87% in patients with CRP < 1.0 mg/dL (14). Our hospital's protocol defines successful treatment of septic arthritis and readiness for discharge as absence of fever, obvious clinical improvement, and down-trending of CRP to < 2.0 mg/dL (laboratory normal reference range: < 0.8 mg/dL).

The senior author noted a lack of consistent adherence to the discharge criterion of <2.0 mg/dL. We sought to examine whether there was an association between discharge of a patient with a CRP > 2.0 mg/dL and treatment failure defined as readmission or reoperation. The purpose of this study was to evaluate the efficacy of a discharge criterion of CRP < 2.0 mg/dL at preventing reoperation and readmission and to identify other potential risk factors for treatment failure of septic arthritis in children.

## Methods

This is a retrospective study of patients who underwent surgical treatment of septic arthritis between January 1, 2007 and December 31, 2017 at a single children's hospital. Patients were identified by International Classification of Diseases (ICD) 9 and 10 diagnostic codes and the relevant surgical current procedural terminology (CPT) codes. Inclusion criteria were children <18 years of age with confirmed septic arthritis of the shoulder, elbow, wrist, hip, knee, or ankle. Septic arthritis was diagnosed based on clinical presentation, the finding of purulent material on joint aspiration or the isolation of a bacterial pathogen from joint fluid or tissue, and laboratory results. Laboratory classification followed Kocher criteria of ESR > 40 mm/h and WBC > 12,000.

Exclusion criteria were patients previously diagnosed with inflammatory or immunosuppressive conditions, patients with post-operative wound infections, concurrent necrotizing soft tissue infection (NSTI), and those with septic arthritis with known adjacent osteomyelitis based on MRI at presentation. For those who did have an MRI, any patients with confirmed concurrent osteomyelitis at admission were excluded, as their clinical course and treatment would be different based on our institutional guidelines from those patients presumed to have only septic arthritis. Patients who later developed osteomyelitis adjacent to the septic joint were included. Patients who transferred into or out of the institution during their inpatient course were excluded.

Age, sex, site of infection, presenting CRP level, date of index surgical debridement, cultured organism, discharge date, antibiotics given, and postoperative CRP levels were recorded by chart review. The cohort of readmitted patients, whether they underwent reoperation or not, was compared to the cohort of patients who were not readmitted. Bivariate tests of associations between potential risk factors and readmission and reoperation were performed. Mann-Whitney tests were used for the quantitative factors and Chi-square tests for the categorical factors. Statistical significance was set at *p* ≤ 0.05 and all confidence intervals at 95%.

## Results

One hundred and eighty-three children were included in the study. Seven (3.8%) were readmitted after hospital discharge for further management, including six requiring reoperation. The demographics of the cohort and descriptive characteristics of the patients' infections are summarized in Tables 1, 2.

**Table 1:** Patient demographics.

	**All patients** **(*n* = 183)**	**Single-admission patients** **(*n* = 176, 96.2%)**	**Readmission patients** **(*n* = 7, 3.8%)**	***p* value**
**Age**				
Mean ± SD	5.5 ± 4.3	5.5 ± 4.4	5.4 ± 3.5	0.804
Range	Newborn−18 years	Newborn−18 years	1–13 years	
**Gender**				
Female	67 (36.7%)	65 (36.9%)	2 (28.6%)	0.653
Male	116 (63.3%)	111 (63.1%)	5 (71.4%)	
**Joint**				
Hip	77 (42.1%)	75 (42.6%)	2 (28.6%)	0.109
Knee	51 (27.9%)	50 (28.4%)	1 (14.3%)	
Ankle/Foot	16 (8.7%)	13 (7.4%)	3 (42.9%)	
Shoulder	27 (14.8%)	26 (14.8%)	1 (14.3%)	
Elbow/Wrist	12 (6.6%)	12 (6.8%)	0	

**Table 2:** Causative organisms in single-admission patients and readmitted patients.

**Organism**	**Number affected**
**Single-admission patients (183)**	
Culture-negative	84
Methicillin-sensitive *Staphylococcus aureus*	52
Group A *Streptococcus*	14
Methicillin-resistant *Staphylococcus aureus*	7
Group B *Streptococcus*	5
Coagulase-negative *Staphylococcus aureus*	5
*Kingella kingae*	5
*Streptococcus pneumoniae*	4
**Readmitted patients (7)**	
Culture-negative	1
*Propionobacterium acnes*	1
Group A *Streptococcus*	1
Methicillin-resistant *Staphylococcus aureus*	1
*Mycobacterium abscessus*	1
Coagulase-negative *Staphylococcus aureus*	1
*Kingella kingae*	1

There were no significant differences in age or sex between the single-admission and readmitted children with septic arthritis. Mean age was 5.4 ± 3.5 years in the readmitted patients and 5.5 ± 4.4 years in the single-admission patients (*p* = 0.804). There were more males than females: 63% vs. 37% in the single-admission patients and 71% vs. 29% in the readmitted patients (*p* = 0.653). Lower extremity joints were most commonly involved in both groups (*p* = 0.109).

**Figure 1:**
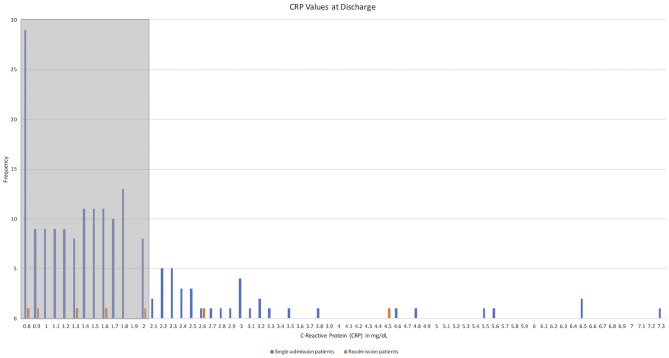
CRP values at discharge.

Mean CRP value on presentation was similar between the two groups: 8.26 mg/dL (± 7.87) in the single-admission patients and 7.94 mg/dL (± 11.26) in the readmitted patients (*p* = 0.430). Length of admission was similar with a mean of 7.3 days (± 5.4 days) for single-admission patients and 6 days (± 3 days) for readmitted patients (*p* = 0.465). Mean CRP at discharge for single-admission patients was 1.71 mg/dL (± 1.07), whereas the value was 1.96 mg/dL (± 1.19) for the readmitted patients (*p* = 0.664). See Figure 1 for a distribution of CRP values at discharge. A total of 48 children (25.9%) had CRP values greater than the suggested 2.0 mg/dL at discharge, but only three of 48 (6.2%) were later readmitted. The mean post-operative day (POD) at readmission was 23.3, with a range of 5–56 days, while mean POD at time of reoperation was 23.5, with a range of 5–57 days.

A total of 95 patients (51.9%) had culture-positive septic arthritis based on aspirate or intra-operative cultures, while 84 (45.9%) were culture-negative. The most common organism was methicillin-sensitive *Staphylococcus aureus* (MSSA) in 52 cases (28.4% of total, 54.7% of culture-positive population), followed by Group A *Streptococcus* (GAS) with 14 cases (7.6% of total) and methicillin-resistant *Staphylococcus aureus* (MRSA) with seven cases (3.8% of total) (see Table 2).

Of the seven readmitted patients, three were culture-negative on discharge; however, two patients' cultures eventually grew an organism (see Table 3). The organisms in the six readmitted patients with positive cultures were Group A *Streptococcus, Kingella kingae*, methicillin-resistant *Staphylococcus aureus, Propionobacterium acnes, Mycobacterium abscessus*, and coagulase-negative *Staphylococcus aureus*. Six of the seven readmitted patients had repeat surgeries, with one patient readmitted for additional IV antibiotics only. Three of the six patients requiring reoperation had adjacent osteomyelitis on MRI at time of readmission. The only patient who remained culture-negative was ultimately diagnosed with juvenile idiopathic arthritis (JIA).

**Table 3:** Patient factors, laboratory values, and imaging results of patients readmitted after initial discharge.

**Age**	**Sex**	**Admission CRP**	**Discharge CRP**	**Readmission CRP**	**Culture result at discharge**	**Culture organism**	**Joint affected**	**POD readmission**	**MRI findings**	**Reoperation POD and procedure(s)**
6	M	3.5	2.0	0.8	+	Coagulase-negative *Staphylococcus aureus*	Hip	11	– for osteomyelitis + for synovitis with associated effusion	POD 11 1. Arthrotomy, R knee 2. Washout of soft tissues 3. Aspiration of knee joint 4. Aspiration femur and tibia
6	M	4.7	4.5	3.0	+	Group A *Streptococcus*	Hip	37	+ femoral osteomyelitis and hypoperfusion + for hip effusion and possible acetabulum osteomyelitis	POD 37 1. Incision and drainage L femur 2. Incision and drainage trochanter neck L proximal femur 3. Incision and drainage with arthrotomy, L hip 4. Aspiration, L hip 5. Aspiration, L knee
3	M	0.2	0.8	0.8	–	*Propionobacterium acnes*	Knee	5	+ for early osteomyelitis in femoral condyle – for joint effusion	POD 5 1. Arthrocentesis R knee and arthrotomy
4	F	2.8	2.6	2.6	–	Culture-negative Note: this patient was ultimately diagnosed with JIA	Foot/ankle	16	– for osteomyelitis in L ankle + right biceps abscess, effusion with rim enhancement in R shoulder joint and elbow joint effusion concerning for osteomyelitis	POD 17 1. IR drainage catheter placement of R arm abscess
1	F	4.3	0.9	1.6	+	*Kingella kingae*	Foot/ankle	NA, no initial I&D	NA	POD 5 (from aspiration) 1. Incision and drainage of L ankle
13	M	35.3	1.6	4.6	+	Methicillin-resistant *Staphylococcus aureus*	Foot/ankle	56	+ for osteomyelitis in distal tibia + for fluid in soft tissues around ankle	POD 57 1. I&D of left tibia with sequestrectomy
5	M	4.8	1.3	Not available	–	*Mycobacterium abscessus*	Knee	15	– for osteomyelitis + for knee effusion	NA, addition of IV antibiotics

## Discussion

Use of CRP values in the diagnosis and management of pediatric septic arthritis is standard of care. Our institutional policy is to discharge patients with septic arthritis only once the CRP level is <2.0 mg/dL, however there is little evidence in the literature to support this. To our knowledge, this is the first study to examine the relationship between CRP value at discharge and readmission/reoperation rates in pediatric patients treated for septic arthritis.

The readmitted and single-admission patients in our study were statistically similar in patient and infection characteristics: age, sex, site of infection, presenting, and discharge CRP levels. These results are consistent with previously reported literature that shows males are more commonly affected than females and that lower extremities account for up to 85% of cases of septic arthritis (3). As in the literature, we did not identify that any particular demographic or patient factor posed a higher risk for readmission or reoperation when excluding immunocompromised patients (3, 12, 13). Readmitted patients did not appear to have any predictive factors; of the readmitted patients, aged 1–13 years old, and each of the six culture-positive patients demonstrated a unique organism (Table 3).

The rate of negative species identification from aspiration or tissue culture was 45.9% in our cohort. This is consistent with the published reports of high rates of culture-negative cases, up to 70%, despite laboratory and clinical findings suggestive septic arthritis (11). Three of the seven readmitted patients were presumed culture-negative at the time of discharge though two ultimately grew an atypical, and commonly more aggressive, causative organism. This finding behooves the surgeon to carefully monitor these patients after discharge.

Our one readmitted patient with persistently negative culture results was ultimately diagnosed with JIA. This patient was included in analysis as they met septic arthritis criteria, as defined above, on admission. The patient's clinical course was consistent with septic arthritis and the patient was only readmitted when outpatient follow-up labs demonstrated elevated CRP and ESR. The patient's MRI demonstrated a soft tissue abscess and rim-enhancing joint effusions. This emphasizes the importance of considering an inflammatory etiology in suspected septic arthritis patients, particularly those who do not show typical response to appropriate septic arthritis treatment and whose cultures are negative. Previous research has demonstrated that while there are no sufficiently reliable predictors for differentiating between septic arthritis and JIA, JIA should be considered in cases of poor disease resolution after antibiotic treatment and joint aspiration (12).

Four of the seven readmitted patients were discharged according to our institutional protocol once CRP was < 2.0 mg/dL, while three had values ≥ 2.0 mg/dL on discharge: 2.0, 4.5, and 2.6 mg/dL on POD 4, 8, and 6, respectively. Three of the readmitted patients had MRIs after readmission but prior to irrigation and debridement confirming either delayed diagnosis or development of osteomyelitis. This may suggest that if serial CRPs fail to downtrend or resume an upward trend, or if patients have a recurrence of fever, pain or local symptoms, children should be carefully evaluated for persistent septic arthritis or adjacent osteomyelitis and may require advanced imaging such as MRI. Since 93.8% of patients discharged with a CRP of >2.0 mg/dL were not readmitted, a strict discharge CRP criterion may be of limited utility. There are no other studies in the literature to date evaluating CRP levels as a criterion for discharge after septic arthritis treatment. These data do not obviate the efficacy of CRP in monitoring as confirmed in previous studies (13, 15).

Our data also suggest the cultured organism is important and may predict the cases of septic arthritis that are more difficult to treat and might be at risk for readmission or reoperation. In the single-admission patients, MSSA was the causative organism in 28.6% of patients, while unusual or resistant bacteria were identified in 25.5% of patients, and samples were culture negative in 45.9%. Of the readmitted patients, two of seven (28.5%) grew *Staphylococcus aureus*, one of which was methicillin-resistant. Three of seven readmitted patients (42.9%) had unusual organisms *(Propionobacterium acnes* isolated from broth only*, Kingella kingae*, and *Mycobacterium abscessus)* and two were in the patients with cultures that became positive only after discharge. Polymerase chain reaction (PCR) may have utility in these readmitted cases allowing for earlier detection of bacteria, and possibly before discharge (16). Bacterial growth of certain organisms such as *Kingella kingae*, MSSA, *Enterococcus faecalis*, and coagulase-negative *Staphylococcus* can be delayed and incubation of synovial fluid cultures for up to 4 days before considering them to be negative has been recommended (17). PCR markedly improves rate of detection of *Kingella kingae* markedly when compared with joint fluid culture (16).

Previous research done by Telleria et al. demonstrated that need for revision surgery was associated with positive blood culture or marked elevation in CRP at presentation or on POD 1–4 (18). However, similar to our observations, that study also demonstrated that certain organisms such as MRSA, *Streptococcus pyogenes*, and *Propionobacterium acnes* are associated with revision surgery, but readmission rates were not examined (18).

This study has several limitations. It is a retrospective study with a small comparative group. The sample size of only seven readmitted patients is small and may not be representative of larger populations. A power analysis was not conducted because our study reports the experience with a protocol in one institution. It could not be known a priori what the readmission rate was so the study could not be powered before it was conducted. *Post-hoc* power analysis has been demonstrated to be of little utility and thus was not performed here (19). This study was conducted at a single children's hospital and therefore patient and bacterial characteristics may not be generalizable to populations in other regions. Patients were operated on by different surgeons and consulted on by different infectious disease specialists. Variability in surgical technique is to be expected, in addition to differences in perioperative management. MRIs were not routinely obtained on admission prior to surgical irrigation and debridement. Concurrent osteomyelitis at admission could have been present in some of our readmitted patients though clinically and radiographically we do not believe this to be the case.

## Conclusions

CRP levels are useful in monitoring treatment efficacy of septic arthritis in children but are not reliable as a discharge criterion to prevent readmission or reoperation. This study suggests the bacterial organism may be more important in predicting treatment success. Atypical and antibiotic-resistant organisms should prompt concern for a potentially more aggressive infection that may require repeat surgical interventions and/or broader antibiotic coverage. If patients are discharged when cultures remain negative, these patients should also be followed closely to identify signs of persistent infection. We recommend determining discharge readiness on the basis of clinical improvement and down-trending CRP values.

## Data Availability Statement

The raw data supporting the conclusions of this article will be made available by the authors, without undue reservation, to any qualified researcher.

## Ethics Statement

The studies involving human participants were reviewed and approved by Institutional Review Board at Seattle Children's Research Institute. Written informed consent to participate in this study was provided by the participants' legal guardian/next of kin. Written informed consent was obtained from the minor(s)' legal guardian/next of kin for the publication of any potentially identifiable images or data included in this article.

## Author Contributions

MB contributed study design and manuscript preparation. LS performed data analysis and manuscript preparation. VB performed statistical analysis.

### Conflict of Interest

The authors declare that the research was conducted in the absence of any commercial or financial relationships that could be construed as a potential conflict of interest.
